# Dynamics of the Zebrafish Skeleton in Three Dimensions During Juvenile and Adult Development

**DOI:** 10.3389/fphys.2022.875866

**Published:** 2022-05-26

**Authors:** Stacy V. Nguyen, Dominic Lanni, Yongqi Xu, James S. Michaelson, Sarah K. McMenamin

**Affiliations:** ^1^ Biology Department, Boston College, Chestnut Hill, MA, United States; ^2^ Biology Department, Vassar College, Poughkeepsie, NY, United States; ^3^ Department of Pathology, Massachusetts General Hospital, Boston, MA, United States

**Keywords:** skeletogenesis, zebrafish, microcomputed tomographic (micro-CT), skeletal anatomy, juvenile development

## Abstract

Zebrafish are a valuable model for normal vertebrate skeletogenesis and the study of myriad bone disorders. Bones grow, ossify and change shape throughout the zebrafish lifetime, and 3D technologies allow us to examine skeletogenic processes in detail through late developmental stages. To facilitate analysis of shape, orientation and tissue density of skeletal elements throughout ontogeny and adulthood, we generated a high-resolution skeletal reference dataset of wild-type zebrafish development. Using microCT technology, we produced 3D models of the skeletons of individuals ranging from 12 to 25 mm standard length (SL). We analyzed the dynamics of skeletal density and volume as they increase during juvenile and adult growth. Our resource allows anatomical comparisons between meristic units within an individual—e.g., we show that the vertebral canal width increases posteriorly along the spine. Further, structures may be compared between individuals at different body sizes: we highlight the shape changes that the lower jaw undergoes as fish mature from juvenile to adult. We show that even reproductively mature adult zebrafish (17–25 mm SL) continue to undergo substantial changes in skeletal morphology and composition with continued adult growth. We provide a segmented model of the adult skull and a series of interactive 3D PDFs at a range of key stages. These resources allow changes in the skeleton to be assessed quantitatively and qualitatively through late stages of development, and can serve as anatomical references for both research and education.

## Introduction

Zebrafish are an efficient and high-throughput model for studying development, and this system is emerging as a powerful tool for skeleton research (Raterman et al., 2020; Tonelli et al., 2020). Zebrafish skeletogenesis is similar in several ways to mammalian skeletal development, and the fish skeleton includes intramembranous and endochondrally ossifying elements (Krane, 2005; Ghayor et al., 2011), as well as both cellular and acellular bones (Weigele and Franz-Odendaal, 2016). The major signaling pathways that regulate skeletal development are highly conserved between mammals and teleosts (Witten and Huysseune, 2009), and zebrafish are a tractable model of vertebrate skeletogenesis with relevance to biomedicine (Hammond et al., 2012). Indeed, numerous studies have leveraged the zebrafish skeleton to investigate skeletal development and homeostasis (e.g. Witten et al., 2001; Crucke et al., 2015; Weigele and Franz-Odendaal, 2016; Machado and Eames, 2017; Parsons et al., 2018). Further, a variety of mutant phenotypes in zebrafish model human bone disorders (Harris et al., 2014; Kwon et al., 2019; Dietrich et al., 2021).

The zebrafish skeleton is comprised of bones that form a dermal skeleton (which includes teeth, scales and fin rays) and an endoskeleton composed of the axial, craniofacial and appendicular elements (Tonelli et al., 2020). Previous work has focused on the sequence of ossification of these bones during early larval development (Cubbage and Mabee, 1996; Bird and Mabee, 2003; Kimmel et al., 2010). However, less is known about skeletal changes during later juvenile and adult stages of development. Several craniofacial bones—including the dermatocranium and infraorbitals—do not become fully ossified until adult stages in zebrafish (Chang and Franz-Odendaal, 2014; Mork and Crump, 2015). Histological stains (e.g., alcian blue and alizarin red) and transgenic reporter lines are valuable tools for imaging and analyzing the dynamic skeleton (Clément et al., 2008; Hammond et al., 2012; Pasqualetti et al., 2012; Rigueur and Lyons, 2014; Bensimon-Brito et al., 2016). More recent technologies—including confocal microscopy, optimal projection tomography and microcomputed tomography (microCT)—allow skeletal elements to be evaluated at high resolution in three dimensions (Bruneel and Witten, 2015; Kanther et al., 2019; Allalou et al., 2020; Bagwell et al., 2020).

Several resources detail the normal anatomical development of the zebrafish skeleton, focusing in particular on larval stages and the initial appearance of different bones. Groups have characterized ossification sequence in the craniofacial skeleton and pectoral girdle (Bird and Mabee, 2003) and the axial skeleton (Cubbage and Mabee, 1996), with a focus on larval stages. Many of the postembryonic stages of development are defined by the ossification of specific skeletal elements (Cubbage and Mabee, 1996; Parichy et al., 2009). FishFace is an online atlas of zebrafish craniofacial development, generated using fluorescent optical projection tomography (Eames et al., 2013). This database serves as a repository of confocal images that capture the development of individual craniofacial elements up to 21 days post fertilization (dpf) (Eames et al., 2013), roughly equivalent to the AR (anal rays) and DR (dorsal rays) stages of larval development according to the postembryonic normal table (Parichy et al., 2009). FishFace also includes an interactive 3D tool for viewing the entire head at three select developmental stages (Eames et al., 2013).

Over the past decade, microCT has served as a powerful tool for assessing phenotypes at a high resolution and in 3D (Charles et al., 2017; Hur et al., 2017). MicroCT has been used to capture the ways in which altered gene function affects skeletal phenotypes in zebrafish (Charles et al., 2017; Hur et al., 2017; Silvent et al., 2017; Caetano-Lopes et al., 2020). While microCT data provide researchers with valuable information, data-rich scans can require large amounts of storage space and access to costly analysis software (Tesařová et al., 2019). In recent years, several developmental atlases have been generated from microCT scans for other models and organs, including a 3D atlas of the developing human embryo and the developing mouse heart (de Bakker et al., 2012; de Boer et al., 2012).

To capture the changes which the zebrafish skeleton undergoes during juvenile and adult development, we generated an accessible skeletal reference from microCT scans of individuals ranging in size from 12 to 25 mm standard length (SL), ranging from J (juvenile) through A (adult) stages (Parichy et al., 2009). We demonstrate the use of this resource to quantify skeletal changes occurring with growth and development. Using this dataset, we examined the morphological changes of vertebrate along the anterior-posterior axis of the vertebral column during juvenile and adult stages. We tested whether density and volume of the skeleton increase with juvenile and adult growth. Further, we asked whether patterns of skeletal density along the anterio-posterior axis of the skeleton shifts with growth. This reference dataset of normal skeletal development can serve as a baseline to which disrupted developmental phenotypes can be compared. Moreover, we anticipate the dataset can be used as an anatomical reference in both educational and research settings.

## Materials and Methods

### Fish Rearing and Measurement

All studies were performed on an approved protocol in accordance with the Boston College Institutional Animal Care and Use Committee (IACUC; Protocol #2020-005). Zebrafish were reared at 28°C on a 14:10 light:dark cycle and fed a diet of marine rotifers and adult pellet food flakes three times a day. Zebrafish were of the genetic background *Tg(tg:nVenus-2a-nfnB)* ^*wp.rt8*^ (McMenamin et al., 2014) and originated from several matings of the same parental breeding stock. Individuals were treated with 1% DMSO at 4 dpf, which does not activate the transgenic nitroreductase system (McMenamin et al., 2014). To ensure that these individuals were representative and that the transgenic background or the DMSO-treatment did not cause gross skeletal mis-patterning, we scanned representative stages from the Tübingen wild-type line for comparison. The two strains were overall comparable morphologically and in terms of relative density (see Supplementary Figure S1).

Capturing individuals at a range of developmental stages requires precise and repeatable methods for measuring development. Days post fertilization is an unreliable measure of developmental progress in zebrafish, particularly during later stages of development (Parichy et al., 2009; McMenamin et al., 2016). We used SL as a proxy for development, and samples were measured both before scanning and in the scans themselves (see Supplementary Figure S2). Although staging according to the postembryonic normal table is likely a more accurate measurement of developmental progress than length (Parichy et al., 2009; McMenamin et al., 2016), we chose to use fixed SL because it is a continuous and quantitative proxy for development which may be easily obtained from scans. On average, most of the 12 mm SL fish were 2 months old; 16 mm SL fish were between 4 and 5 months age and 24 mm SL fish were 6–9 months old when fixed. The sex of the individuals was also recorded when it was possible to discern sex, starting approximately around 17.5 mm SL (Supplementary Figure S3).

### MicroCT Scanning and Reconstruction

Fish were euthanized by MS-222, and fixed in 4% paraformaldehyde for 24 h. SL was measured in fixed samples according to Parichy et al. (2009) before scanning, and was also measured digitally in the scans themselves (see Supplementary Figure S1). Fish samples shrink slightly during the fixation process; note that the reported fixed SL values may be converted to corresponding “fresh” SL by adding 0.29 mm (Parichy et al., 2009). A total of 62 specimens were scanned, ranging from 12 to 25 mm SL with a minimum of one scan for every half millimeter. Fixed specimens were placed in low-density foam molds and inserted into either a 1.5 ml centrifuge tube (for specimens 12–14 mm SL) or a 15 ml conical tube (for specimens >14 mm SL). Scans were performed on a SkyScan 1275 high resolution microCT system (Bruker, Kontich, Belgium) at a scanning resolution of 10.5 μm with an x-ray source voltage of 45 kV and current of 200 mA. Projection images were generated over 360° with a 0.1° rotation step and 6 averaging frames. Thresholding, ring artifact reduction, and beam hardening corrections were consistent across all scans during reconstruction using NRecon (Bruker, Kontich, Belgium).

### Quantifications and Segmentation

Cross section images were generated in the open source software 3D slicer (Kikinis et al., 2014). Vertebral diameter measurements were taken using Amira 6.5 (Thermo Fisher Scientific FEI, Hillsboro, Oregon, United States) using the orthoslice module to view the transverse cross section and the canal width was quantified with the 2D measurement tool, measuring the diameter of the vertebral canals of all rib bearing vertebrae in zebrafish at four representative sizes (12, 16, 20, and 24 mm SL). Multi-level modeling was performed with pairwise post-hoc analysis to determine significant differences in vertebral diameters at each vertebra among the four standard length sizes. These measurements could alternatively be made using 3D Slicer.

Relative density heatmaps were generated with the volume rendering module and physics load transfer function in Amira with a threshold range of 20–120. Mean gray value was also used to show relative density between scans. Mean gray value was calculated from imported cross section slices using the measurement tool in ImageJ (Version 1.8.0_172, National Institutes of Health, Bethesda, Maryland, United States). Volume measurements were taken with the Material Statistics module in Amira.

Individual bones were segmented using the Segmentation Editor in Amira 6.5 (Thermo Fisher Scientific FEI, Hillsboro, Oregon, United States). Briefly, the entire scan volume was loaded into the program and a pixel threshold was determined to differentiate bone from soft tissue. The lasso tool was then used to select the corresponding pixels of a specific skeletal element and added to the appropriate material label. Segmented bones include the basibrachials, branchial arches, basioccipital, dentary, dermatocranium, ectopterygoid, exocciptal, entopterygoid, hyoid, hyomandibula, infraorbital, interopercle, kinethmoid, lateral ethmoid, maxilla, metapterygoid, opercle, orbitosphenoid, pharyngeal jaws, premaxilla, preopercle, parasphenoid, quadrate, supracleithrum, supraoccipital, subopercle, and supraorbital. Pearson correlation coefficients were calculated to show the correlation between density or volume and SL.

### Interactive 3D PDFs

3D models of the microCT reconstructed scans were generated in Amira 6.5 using the Segmentation Editor and Generate Surface module (Thermo Fisher Scientific FEI, Hillsboro, Oregon, United States). Meshes were simplified using MeshLab (Cignoni et al., 2008; Callieri, 2013). These models were converted to .u3d files and imported as interactive 3D PDF using Acrobat Pro DC (Version 2021.005.20058, Adobe Inc., San Jose, CA, United States).

## Results

### microCT Scan Data in Two Formats

Whole, raw microCT scans for individuals from every half mm SL are available for download (see Table 1; MorphoSource project URL https://www.morphosource.org/projects/000415918?locale=en). When multiple scans were available for each size category (see Supplementary Figure S3 and Supplementary Table S1), we selected the highest quality scan for upload to MorphoSource. Additionally, individuals of four representative sizes (12, 16, 20, and 24 mm SL) were used to generate 3D PDFs (see Table 1; Supplementary Figures S5–S8). These interactive PDFs can be viewed with any standard PDF viewer, including Adobe Acrobat Reader (Adobe Inc., San Jose, CA, United States). These 3D PDFs allow users to turn, rotate, and zoom in to the embedded 3D models.

**TABLE 1:** Categories of sizes, sample numbers and scan ID. Full details of each individual may be found in Supplementary Table S1.

SL category (mm)	Number of individuals scanned	MorphoSource ID of representative individual	3D PDF of representative individual
12	2	000415877	Supplementary Figure S5
12.5	1	000416098	-
13	2	000416108	-
13.5	1	000416117	-
14	1	000416167	-
14.5	1	000416187	-
15	1	000416194	-
15.5	2	000416225	-
16	1	000416236	Supplementary Figure S6
16.5	1	000416257	-
17	3	000416263	-
17.5	2	000416291	-
18	1	000416305	-
18.5	1	000416322	-
19	2	000416327	-
19.5	4	000416332	-
20	5	000416337	Supplementary Figure S7
20.5	5	000416342	-
21	5	000416347	-
21.5	5	000416357	-
22	2	000416367	-
22.5	4	000416377	-
23	1	000416382	-
23.5	1	000416387	-
24	3	000416395	Supplementary Figure S8
24.5	2	000416402	-
25	1	000416412	-

### Anatomical Measurements From microCT Cross Sections

**FIGURE 1:**
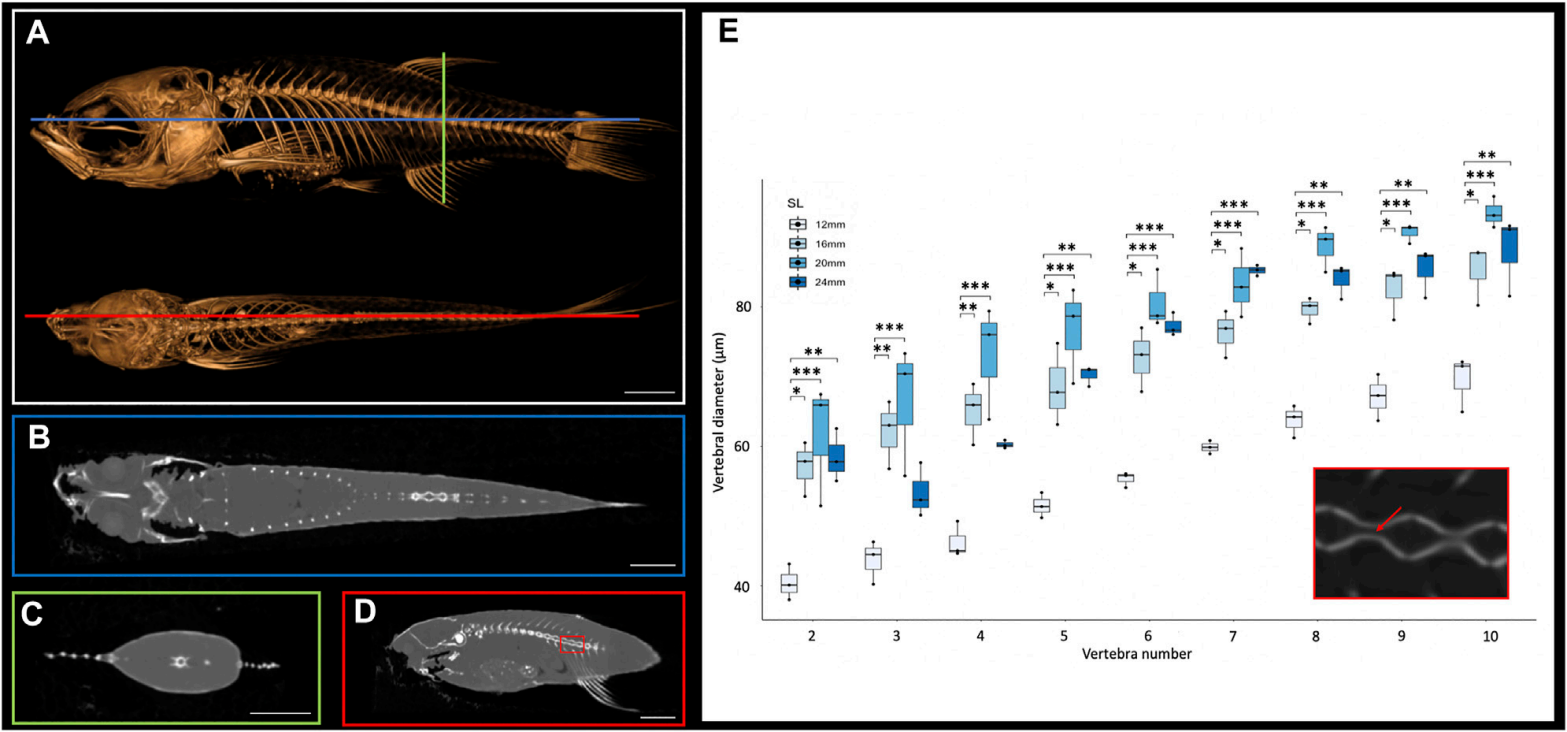
Cross sections of microCT scans visualized in 3D Slicer. **(A)**, Surface rendering of 24 mm SL adult fish with lateral (top) and dorsal (bottom) views with coronal (blue), transverse (green), and sagittal (red) axes indicated. **(B)**, Coronal cross section image. **(C)**, Transverse cross section image. **(D)**, Sagittal cross section image. **(E)**, Quantification of vertebral canal width of 3 individual 24 mm SL adult fish; each individual differentiated with different shapes (circle, square, triangle). Inset shows higher resolution image through vertebrate (corresponding to the boxed detail in panel D). Red arrow indicates a canal from which interior width was measured. Scale bars, 2 mm.

The small size of the zebrafish can pose a barrier to measuring small anatomical elements in 3D. However, microCT technology allows visualization and analysis of elements of interest. MicroCT scans generate cross sections that can be accessed using a variety of programs such as DataViewer (Bruker, Kontich, Belgium), Amira (Thermo Fisher Scientific FEI, Hillsboro, Oregon, United States) or ImageJ (National Institutes of Health, Bethesda, Maryland, United States). Any of these programs will allow a user to scroll through the stacks of cross-section images from the scans in any anatomical plane (e.g., see Figures 1A–D). These cross-sections capture details at a resolution of 10.5 μm, which allows anatomical measurements even in relatively small bones. To test these types of measurements, we focused on the morphological changes of vertebrae along the anterio-posterior axis. We examined sagittal cross sections (as in Figure 1D) from scans of adult zebrafish at four representative sizes (12, 26, 20 and 24 mm SL), measuring the diameter of the vertebral canal of vertebrae 2 through 10 (the rib-bearing vertebrae; Figure 1E). These widths increase markedly in more posterior vertebrae (Figure 1E).

### Zebrafish Skeletons Increase in Density and Volume Throughout Juvenile and Adult Growth

**FIGURE 2:**
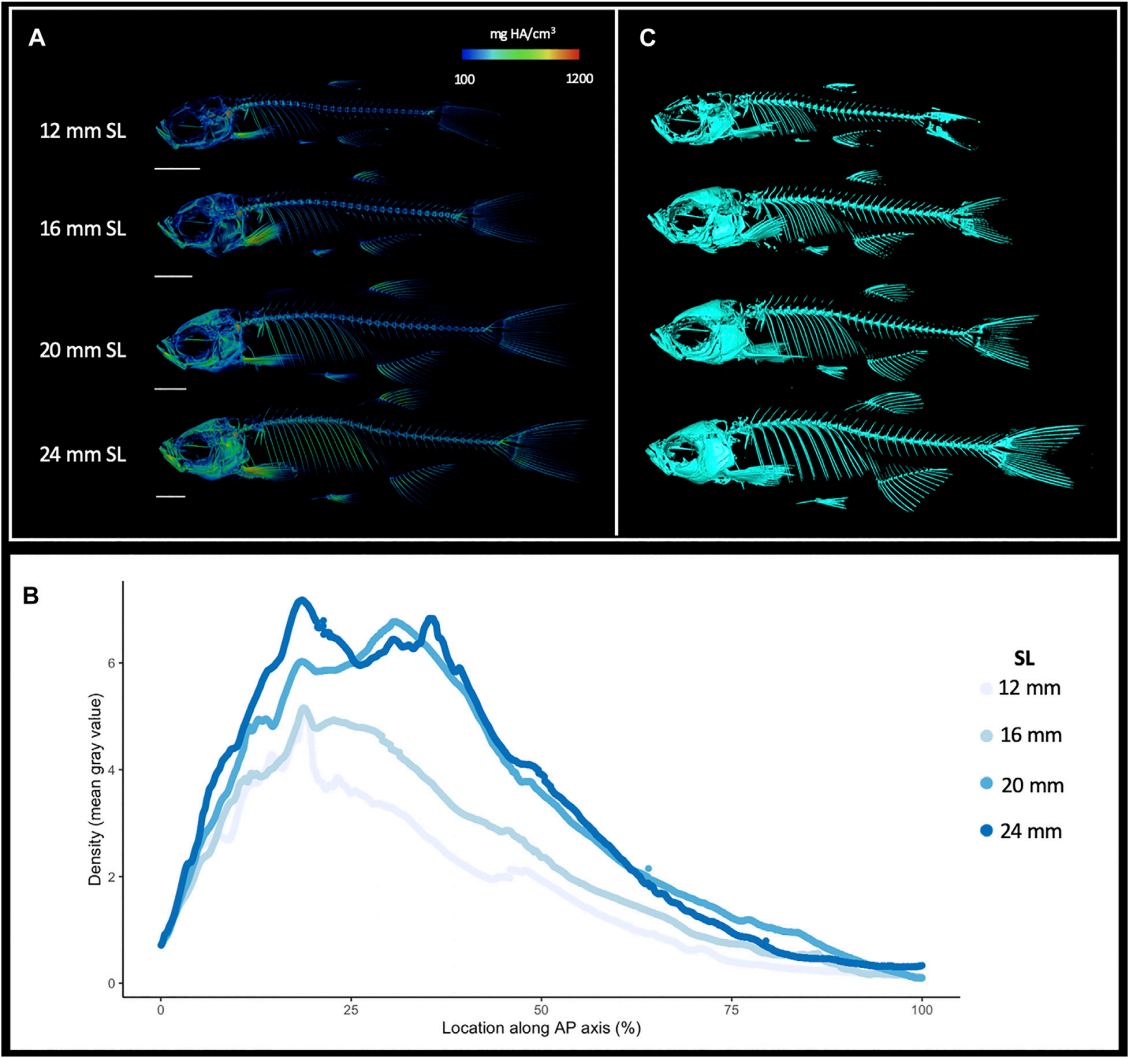
Increasing skeletal density and volume with linear growth. **(A)**, Relative density renderings of skeletons from zebrafish at four different sizes (12, 16, 20, and 24 mm SL). Warmer colors indicate higher density regions. **(B)**, Average density of zebrafish skeleton along the body length of individual zebrafish at four sizes. **(C)**. Volume renderings of zebrafish at four sizes. Scale bars, 2 mm.

**FIGURE 3:**
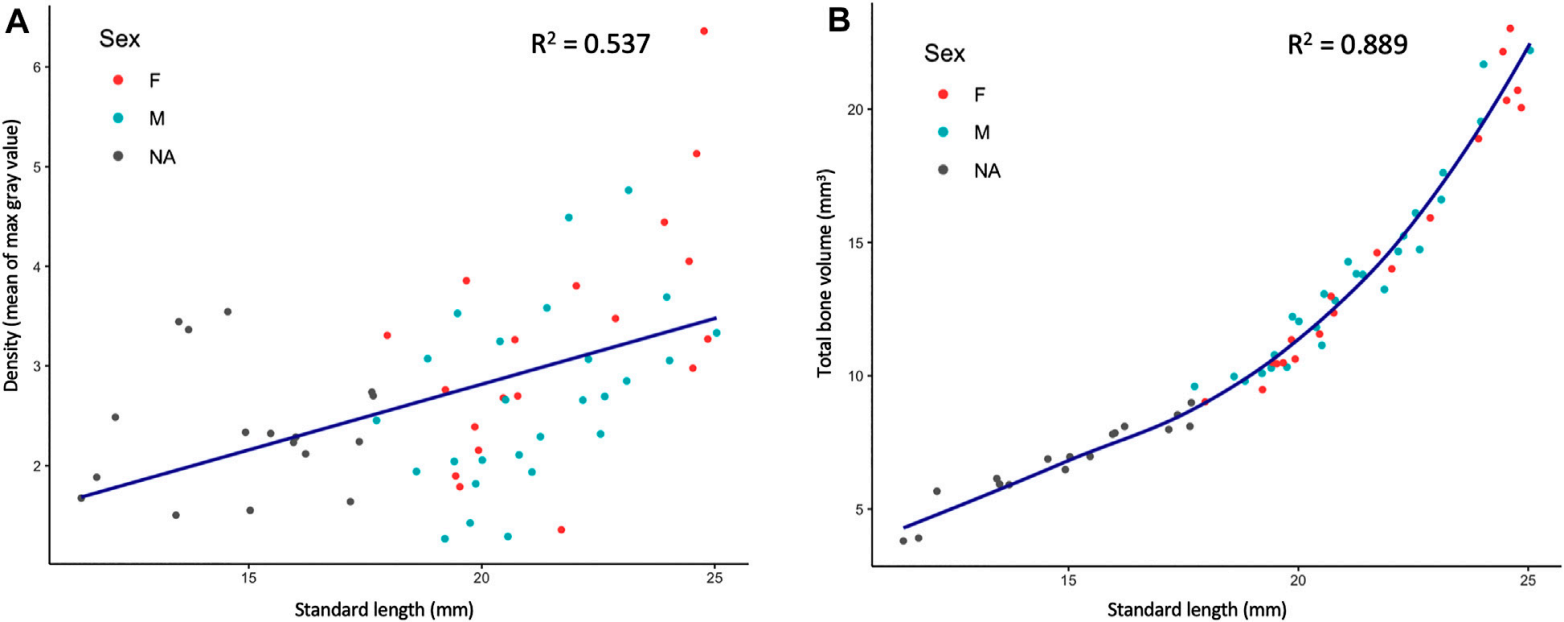
Quantified skeletal density and volume relative to linear growth. **(A)**, Bone density relative to SL. **(B)**, Total bone volume relative to SL. Bone volume calculated from all cross sections of scan.

MicroCT datasets can be used to determine relative density. Our samples were all scanned under consistent parameters, so density can be directly compared between scans. We hypothesized that overall skeletal density would continue increasing throughout stages of adult growth. Indeed, density (as measured in mean grey values) increased markedly with increased size; regions of increasing density were particularly notable in the dermatocranium, ribs, and hypural complex (Figure 2A, Supplementary Videos S1, S2). Quantifying overall density of the skeleton as a function of body size (SL), we found that relative density increases roughly linearly throughout juvenile and adult development (Figure 3A). We next asked how density was distributed along the anterio-posterior axis of the skeleton, and whether such patterns change with growth. We found that density was highest in anterior regions of the body, corresponding to the craniofacial skeleton (Figure 2B). The high density of the head corresponds to the many plate bones in this region. The head also contains three pairs of otoliths; these dense, highly mineralized bony elements are used for hearing and vestibular function (Vasconcelos-Filho et al., 2019), and contribute to the overall density of the head. We note that while maximum density increases in increasingly large individuals, the distribution of density across the skeleton remains largely consistent (Figure 2B). Overall, we also see the same patterns of density distribution when comparing density to distance from the anterior portion of the zebrafish, and when normalizing density to the proportion of body length (Supplementary Figure S4).

**FIGURE 4:**
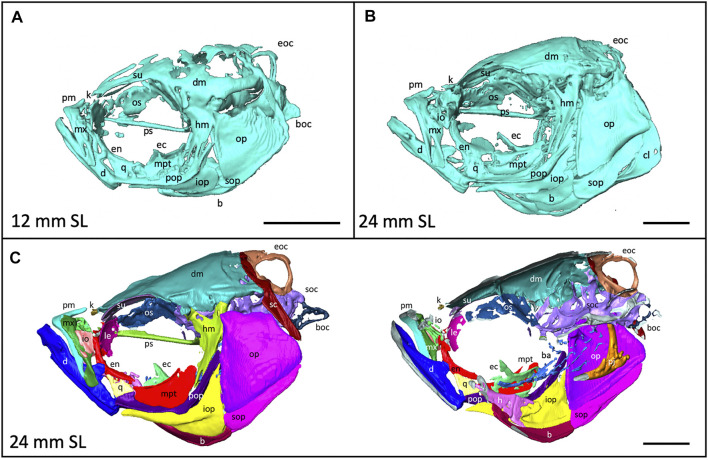
Anatomy of the craniofacial skeleton. **(A)**, Volume rendering of the skull of a 12 mm SL zebrafish and **(B)**, a 24 mm SL zebrafish. **(C)**, Lateral view of skull of 24 mm SL zebrafish with segmented bones (left). Cross section of lateral view reveals some internal elements (right). b, basibrachials; ba, branchial arches; boc, basioccipital; d, dentary; dm, dermatocranium; ec, ectopterygoid; eoc, exocciptal; en, entopterygoid; h, hyoid; hm, hyomandibula; io, infraorbital; iop, interopercle; k, kinethmoid; le, lateral ethmoid; m, maxilla; mpt, metapterygoid; op, opercle; os, orbitosphenoid; pj, pharyngeal jaws; pm, premaxilla; pop, preopercle; ps, parasphenoid, q, quadrate; sc, supracleithrum; soc, supraoccipital; sop, subopercle, su, supraorbital. Scale bars, 1 mm.

MicroCT scans can be used to calculate the volume of tissues within a specified density range. Volumetric renderings of the skeleton highlighted the new appearance of bones in the skull and fins as fish continue to grow (Figure 2C; also see Figures 4A,B). We further quantified the changes in overall skeletal volume, finding a roughly exponential increase in bone volume (a 3D measurement) with linear fish growth (a 2D measurement; Figure 3B).

### Segmentation of Individual Bones Captures Shapes at a Fine Scale

**FIGURE 5:**
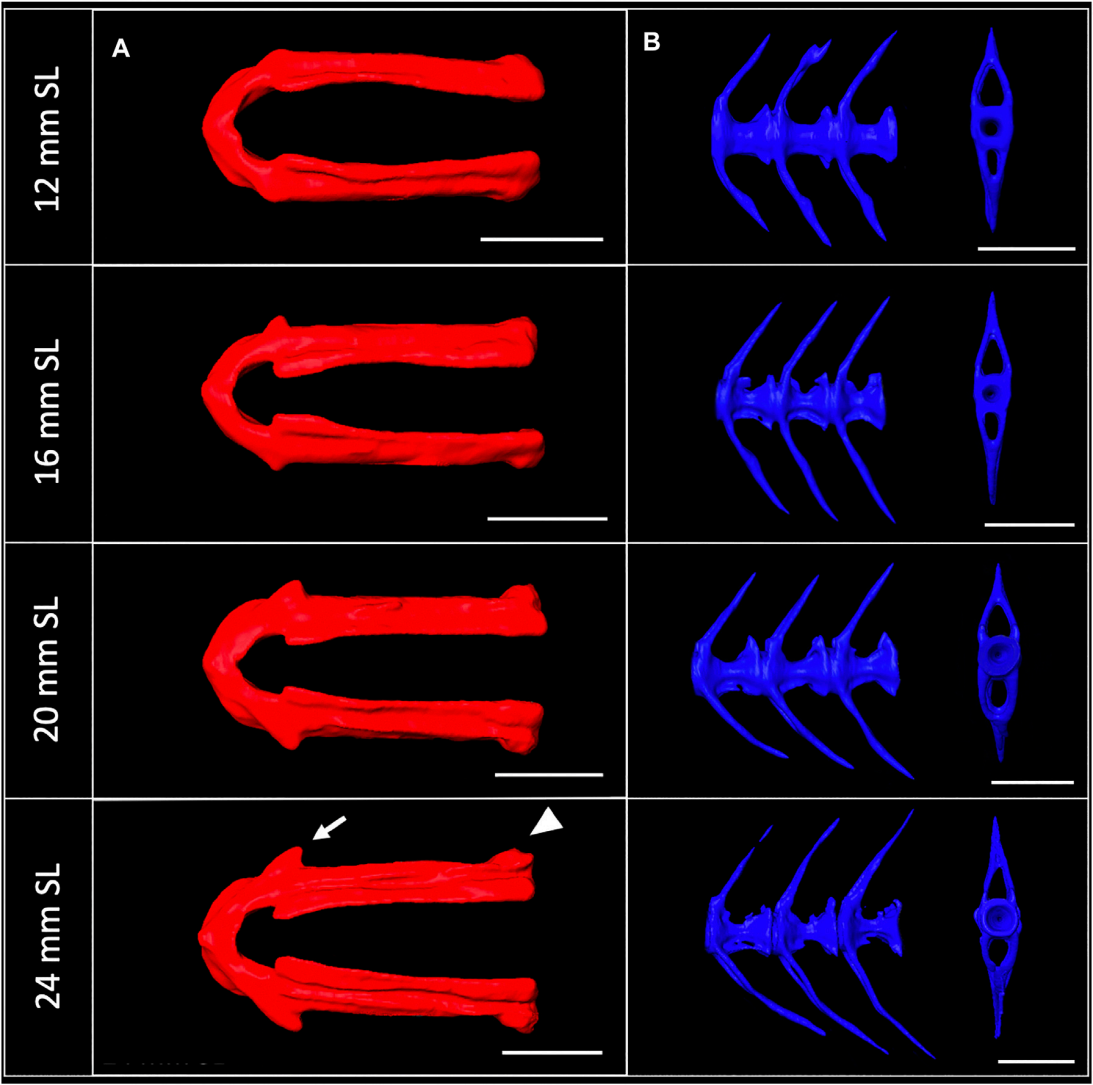
Shape change of the lower jaw and caudal vertebrae. **(A)**, Segmented lower jaws from 12, 16, 20, and 24 mm SL individuals, viewed from the ventral perspective. In the largest individuals, note the pronounced anguloarticular prominence (arrow) and posterior end of lower jaw (arrowhead). **(B)**, Segmented first three caudal vertebrae from 12, 16, 20 and 24 mm SL individuals, viewed from a lateral perspective. Scale bars, 0.5 mm.

3D models can be digitally segmented into individual elements. We segmented an adult skull into the 74 component bones. This segmented model captures the association of each element in 3D space and captures the anatomy of the adult craniofacial skeleton (Figure 4C). After segmentation, bones can be examined individually. To visualize how an individual bone changes shape as development progresses, we “virtually dissected” the lower jaws and caudal vertebrae from fish at a range of sizes (Figures 5A,B). We note that as adult zebrafish continue to grow, the anguloarticular prominence of the lower jaw becomes considerably more pronounced and the posterior end of the jaw widens (Figure 5A), while the caudal vertebrae do not undergo significant shape change during juvenile and adult development (Figure 5B).

## Discussion

The ability to capture shape changes in the skeleton at a fine scale is a powerful technique now being applied to developing organisms. The sensitivity of microCT technology makes it a powerful tool to examine subtle shape differences across developmental stages. In addition to capturing shape, microCT data can be used to quantify density and volume of skeletal elements. Isolating individual elements by segmentation can provide detailed information about spatial orientation and relationships between bones within the skeleton. Using consistent microCT settings at a range of developmental stages offers the ability to track changes in bone composition and morphology across development.

We generated a skeletal reference that allows assessment of skeletal morphology and composition throughout juvenile and adult development in zebrafish. Using this dataset, we showed that total bone volume and density progressively increase even during late stages of development. Additionally, we demonstrate that numerous skeletal elements continue to progressively grow and change shape during juvenile and adult growth, continuing into reproductive maturity. These results emphasize the importance of recording and matching SL between individuals. For the purposes of skeletal research, it is not sufficient to consider all “adult” zebrafish equivalent to one another: sizing and staging should be carefully considered.

This dataset contributes to a growing body of resources for zebrafish researchers, and may be used to examine bone shape during juvenile and adult development at a high resolution. In addition to the interactive 3D PDFs (Supplementary Figures S5–S8), all of the raw data from the microCT scans have been uploaded and made available online at MorphoSource, a repository for 3D data (Boyer et al., 2016) (see Table 1). These scans can be processed using Amira or 3D Slicer (Kikinis et al., 2014), which is open-source. Although here focus our analyses in this manuscript on the skeleton, users of the downloadable raw scans can change the thresholds to visualize and analyze other organs and systems, including the scales, liver and heart across late developmental stages.

Quantitative and qualitative assessment of the scans can highlight regions of the skeleton that are particularly dynamic during late stages of development: e.g., the dermatocranium—which increases in density (see Figure 2A), and the lower jaw—which changes in shape (see Figure 5A). These shifts can inform experimental design by suggesting specific anatomical regions for quantitative focus. Further, the labeled segmented scans (Figure 4) serve as a craniofacial anatomical reference in identifying skeletal elements.

For researchers using zebrafish as a model for skeletal disease, this reference can serve as a normal baseline to which aberrant skeletons can be compared in detail, in terms of morphology, density and skeletal volume. The reference provides a developmental framework for assessing disrupted phenotypes, allowing researchers to assess whether a model of interest shows skeletogenic processes that are accelerated or retarded relative to size. This developmental framework can assist researchers in selecting appropriate body size ranges to evaluate, and can add developmental context even when wild-type individuals (e.g., vehicle controls or non-mutant siblings) are analyzed side-by-side with a disease model. Finally, dynamic processes disrupted in a disease model can be compared to the normal rates of ossification and skeletal change established by this reference.

## Data Availability

The original contributions presented in the study are included in the article/Supplementary Materials, further inquiries can be directed to the corresponding author.

## Supplementary Material

Supplementary Figure 1 DMSO-treated zebrafish have similar skeleton shape and bone density compared to wild-type zebrafish. **(A)**, Relative density renderings of skeletons from zebrafish of original dataset at four different sizes (12, 16, 20, and 24 mm SL) **(B)**, Relative density renderings of skeletons from wild-type zebrafish at the same four sizes (12, 16, 20, and 24 mm SL).

Supplementary Figure 2 Manually measured standard lengths are closely correlated to digitally measured standard lengths. Digital length plotted against manual length for all individuals in the developmental skeletal reference dataset.

Supplementary Figure 3 Histogram showing size distribution of samples scanned. Each dot represents a single individual; orange dots represent female fish and green dots represent male fish. Grey dots indicate fish that were too immature to be accurately sexed.

Supplementary Figure 4
**(A)**, Average density of individual slices along the anterio-posterior axis of individual zebrafish at four sizes. **(B)**, Normalized density plotted against the proportion of the body length.

Supplementary Figure 5 Interactive PDF of 3D model of 12 mm SL zebrafish whole head (top) and whole body (bottom) scans.

Supplementary Figure 6 Interactive PDF of 3D model of 16 mm SL zebrafish whole head (top) and whole body (bottom) scans.

Supplementary Figure 7 Interactive PDF of 3D model of 20 mm SL zebrafish whole head (top) and whole body (bottom) scans.

Supplementary Figure 8 Interactive PDF of 3D model of 24 mm SL zebrafish whole head (top) and whole body (bottom) scans.

Supplementary Video 1 Density scans of the whole zebrafish skeleton ranging from 12 mm to 25 mm SL.

Supplementary Video 2 Colored density scans of zebrafish craniofacial skeleton ranging from 12 mm to 25 mm SL. Warmer colors indicate higher density regions.

Supplementary Table 1 Information about every individual scanned. Note that smaller individuals could not be accurately sexed. For individual scans available on MorphoSource, the ID is given in the last column.
