# Supplementary figures and images for: Dynamics of the Zebrafish Skeleton in Three Dimensions During Juvenile and Adult Development
Source: Front Physiol. 2022 May 26;13:875866. doi: 10.3389/fphys.2022.875866 (PMC9204358; doi:10.3389/fphys.2022.875866)

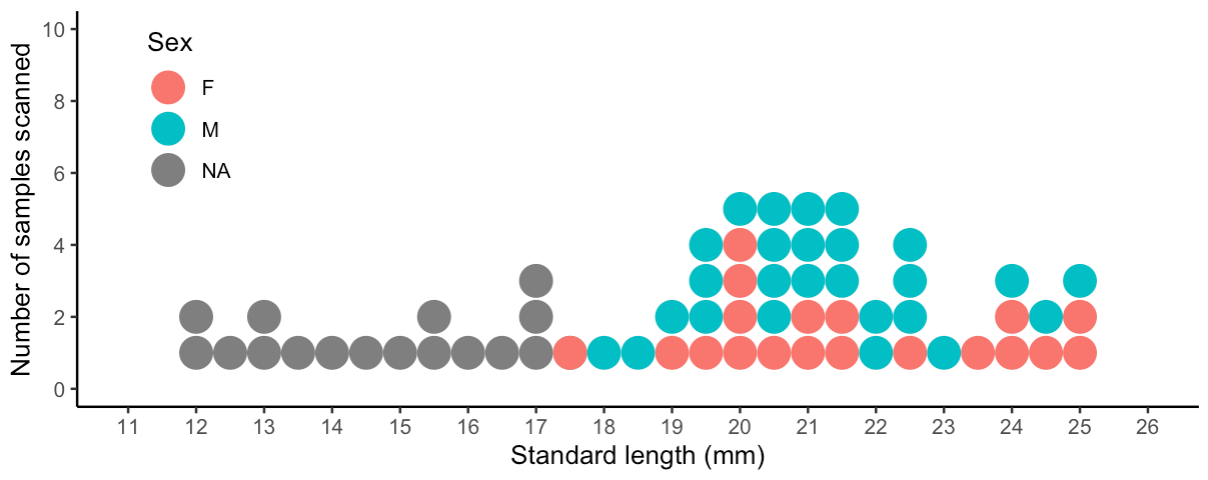

Supplement: Supplementary file 1 [file Image3.TIFF]

# Zebrafish Developmental Atlas

## Interactive 3D PDF

Select a view:

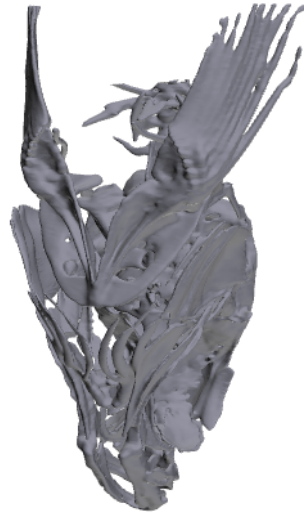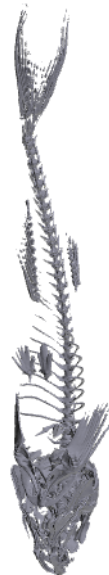

Select a view:

Supplement: Supplementary file 2 [file DataSheet2.PDF]

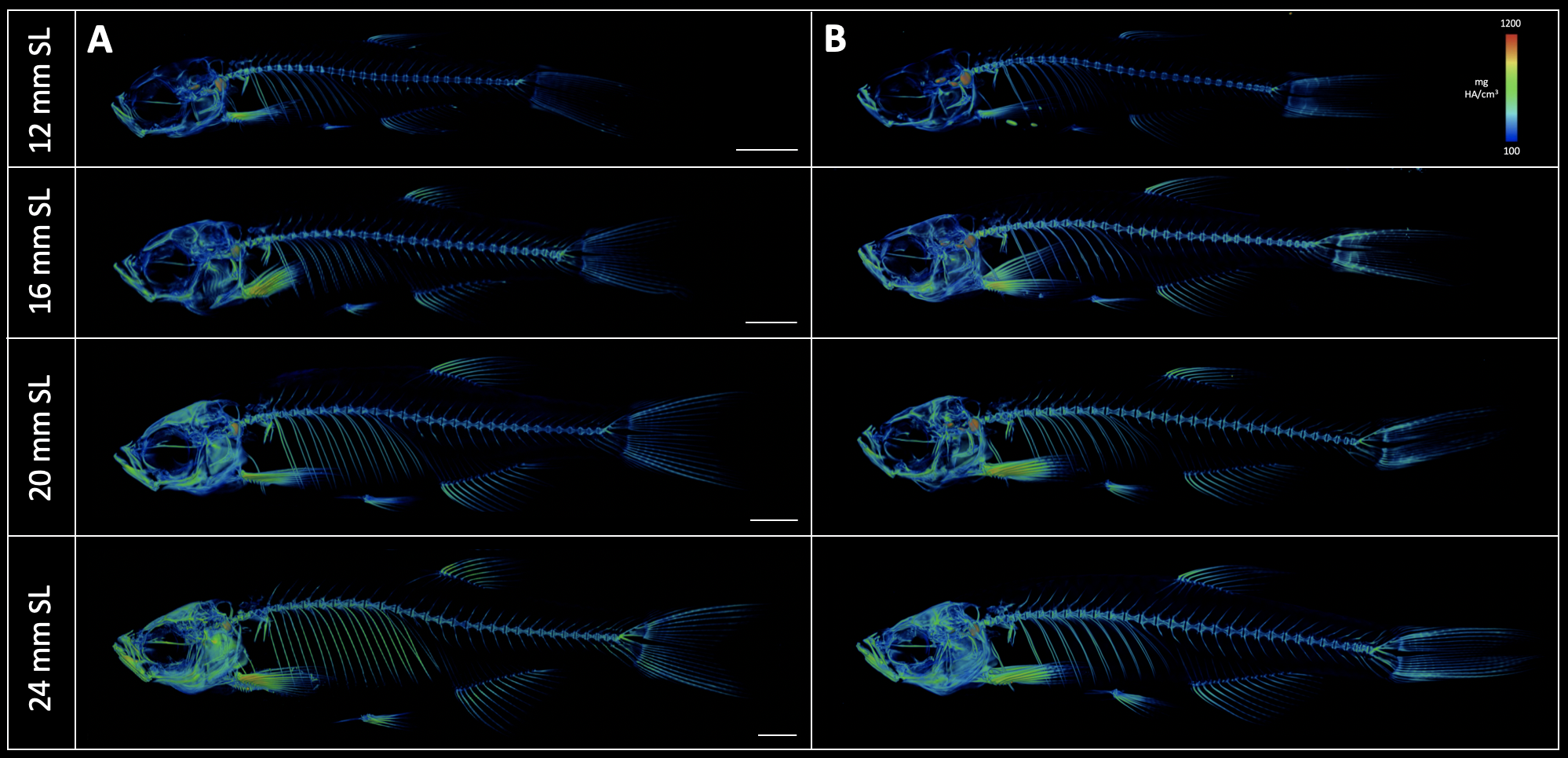

Supplement: Supplementary file 3 [file Image1.TIFF]

# Zebrfish Developmental Atlas

## Interactive 3D PDF

Select a view:

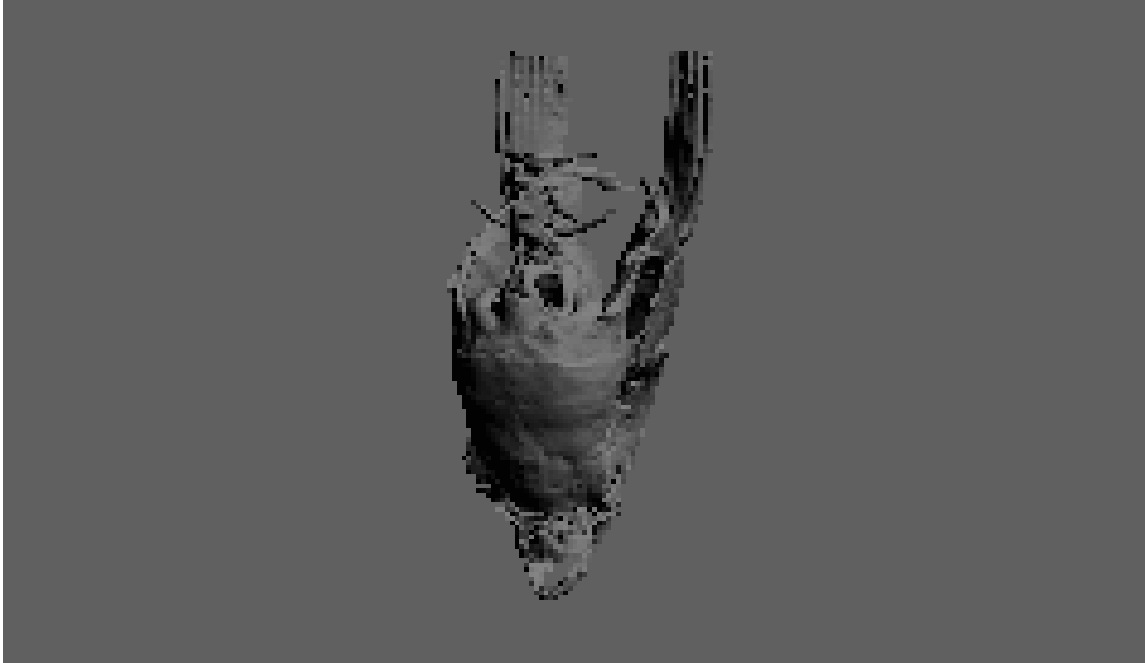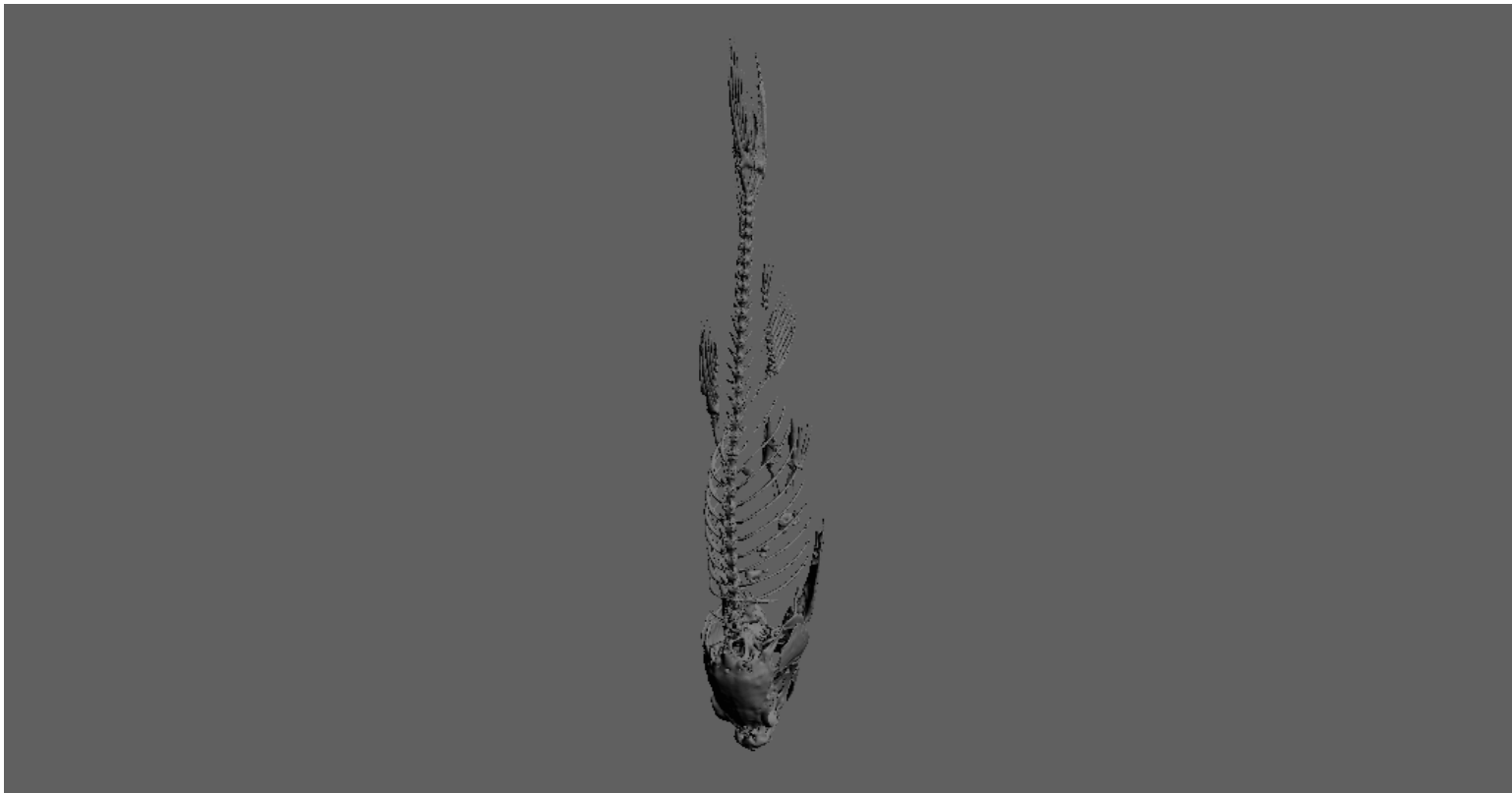

Select a view:

Supplement: Supplementary file 4 [file DataSheet4.PDF]

# Zebraphish Developmental Atlas

## Interactive 3D PDF

Select a view:

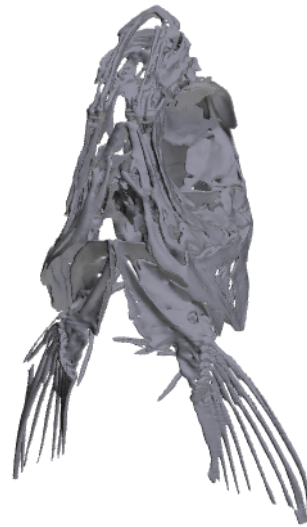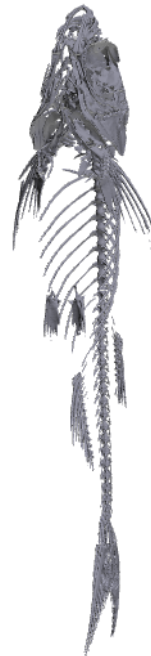

Select a view:

Supplement: Supplementary file 6 [file DataSheet3.PDF]

# Zebrafish Developmental Atlas

## Interactive 3D PDF

Select a view:

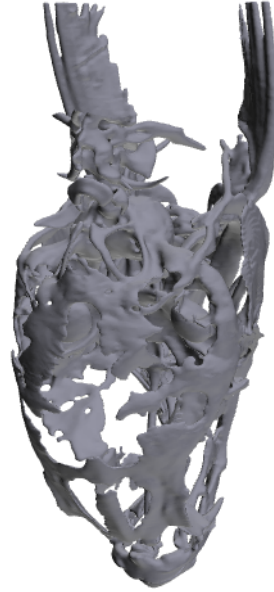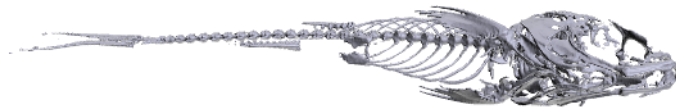

Select a view:

Supplement: Supplementary file 7 [file DataSheet1.PDF]

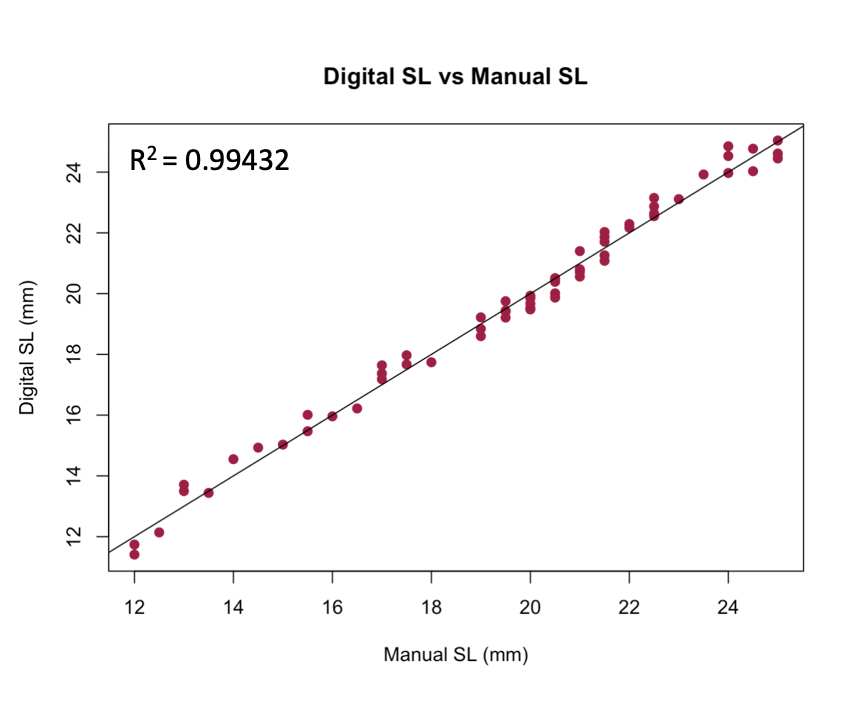

Supplement: Supplementary file 8 [file Image2.TIFF]

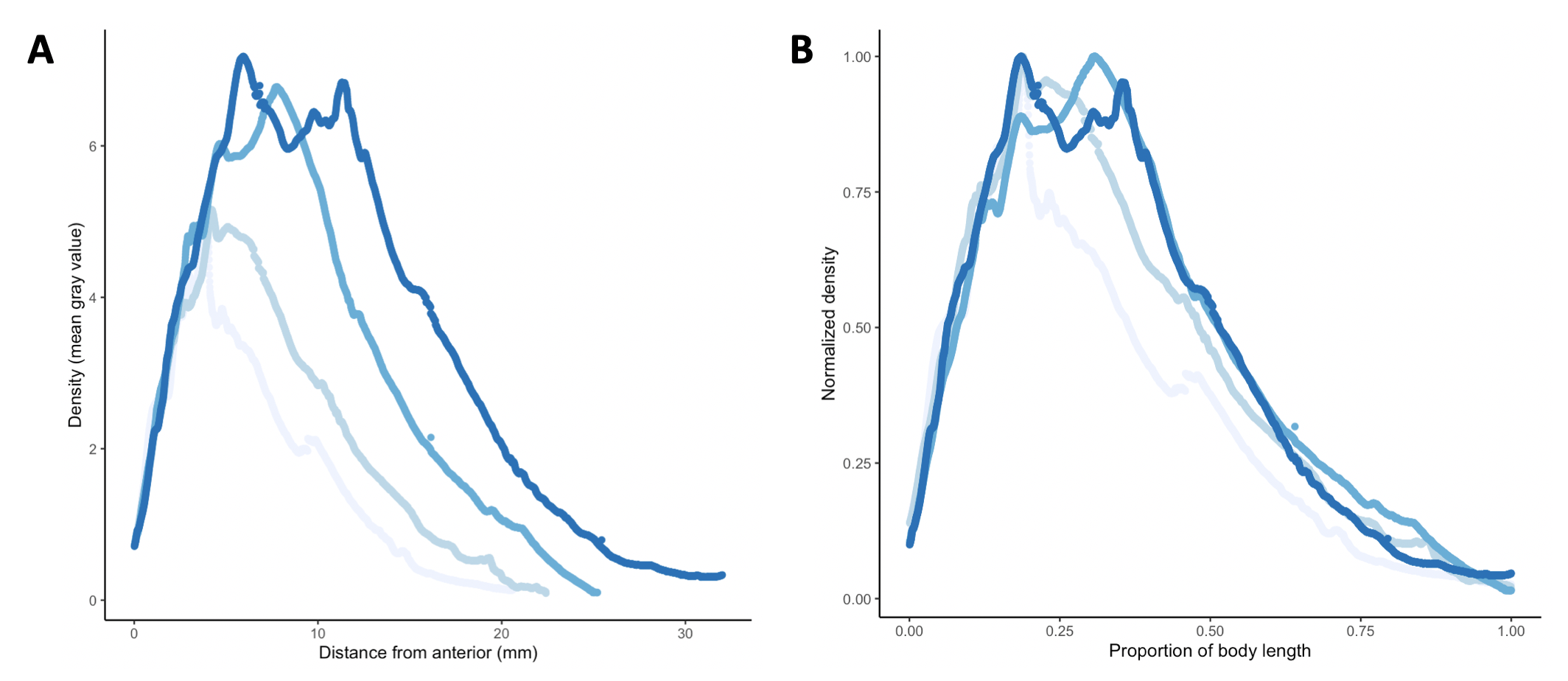

Supplement: Supplementary file 9 [file Image4.TIFF]
